# Metformin Treatment Among Men With Diabetes and the Risk of Prostate Cancer: A Population-Based Historical Cohort Study

**DOI:** 10.1093/aje/kwab287

**Published:** 2021-12-10

**Authors:** Laurence S Freedman, Nirit Agay, Ruth Farmer, Havi Murad, Liraz Olmer, Rachel Dankner

**Keywords:** glucose-lowering medications, inverse probability weighting, marginal structural models, metformin, prostate cancer, time-dependent confounding, type 2 diabetes

## Abstract

There is conflicting evidence regarding the association between metformin treatment and prostate cancer risk in diabetic men. We investigated this association in a population-based Israeli cohort of 145,617 men aged 21–89 years with incident diabetes who were followed over the period 2002–2012. We implemented a time-dependent covariate Cox model, using weighted cumulative exposure to relate metformin history to prostate cancer risk, adjusting for use of other glucose-lowering medications, age, ethnicity, and socioeconomic status. To adjust for time-varying glucose control variables, we used inverse probability weighting of a marginal structural model. With 666,553 person-years of follow-up, 1,592 men were diagnosed with prostate cancer. Metformin exposure in the previous year was positively associated with prostate cancer risk (per defined daily dose; without adjustment for glucose control, hazard ratio (HR) = 1.53 (95% confidence interval (CI): 1.19, 1.96); with adjustment, HR = 1.42 (95% CI: 1.04, 1.94)). However, exposure during the previous 2–7 years was negatively associated with risk (without adjustment for glucose control, HR = 0.58 (95% CI: 0.37, 0.93); with adjustment, HR = 0.60 (95% CI: 0.33, 1.09)). These positive and negative associations with previous-year and earlier metformin exposure, respectively, need to be confirmed and better understood.

## Abbreviations

CI: confidence interval
DDD: defined daily dose
GLM: glucose-lowering medication
HbA1c: hemoglobin A1c
HR: hazard ratio
IPW: inverse probability weighting
MSM: marginal structural model
OR: odds ratio

There is conflicting evidence regarding the effect of metformin therapy on prostate cancer risk. Recent meta-analyses (1–3) of observational studies found no clear evidence of a previously hypothesized protective association (4–7). Nevertheless, analyzing observational study data addressing this question is fraught with pitfalls (8) that, in turn, can influence meta-analysis results, so the question remains open. The high prevalence of diabetes, widespread use of metformin treatment for diabetes, and relatively high incidence of prostate cancer make this question important.

We describe here analysis of a population-based cohort study of patients diagnosed with diabetes, aimed at addressing this question. Important features of our analysis are the use of Cox regression with time-dependent covariates describing metformin treatment history (9) and inverse probability weighting (IPW) of marginal structural models (MSMs) (10). MSM analysis addresses bias arising in Cox regression when a time-varying treatment is modified in response to a time-varying marker—here, hemoglobin A1c (HbA1c) or blood glucose level—that is itself associated with the disease outcome, prostate cancer.

## METHODS

### Data source and study population

The data for this study were obtained from the electronic database of Clalit Health Services (Tel Aviv, Israel), the largest health maintenance organization in Israel, insuring 4.3 million people and comprising a representative 53% of the total population. The database is known to be of high quality and has been the source of many research reports (9, 11–14). Available data comprise a range of clinical measures, including blood glucose and HbA1c levels, and sociodemographic information such as age, socioeconomic status (determined by locality of the Clalit clinic: low, medium, or high SES, or missing data (2.7%)), and ethnicity (determined by country of birth: Ashkenazi Jew (born in Russia, Eastern Europe, Europe, the United States, or South Africa); Sephardic Jew (born in North Africa or the Middle East); Yemenite; Ethiopian or Central African Jew; Israeli-born Jew (when first generation in Israel, the mother’s country of birth determined ethnicity); or Israeli Arab). Data on dispensation of medications are also available.

For this study, the database was linked to the Israel Cancer Registry. Registration of cancer diagnoses is mandated by law in Israel, and the registry reports 97% coverage of solid tumors and 88% coverage of hematological cancers that are diagnosed in Israel (15).

**Figure 1:**
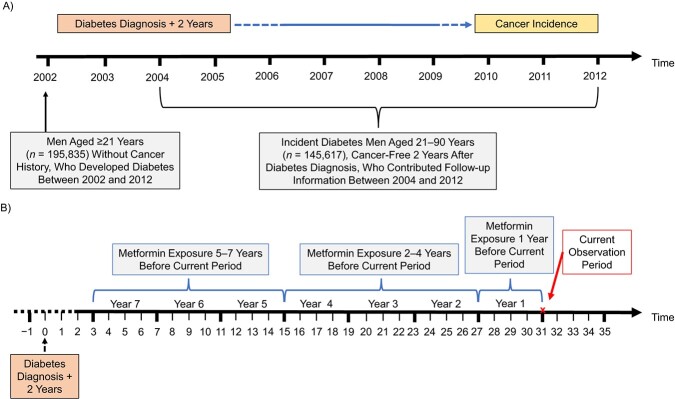
Time frame of a study on metformin treatment among diabetic men and prostate cancer risk, Israel, 2002–2012. A) Calendar time; B) Cox model time frame, months.

The study population consisted of men aged 21–89 years who were newly diagnosed with diabetes in 2002–2012 (see Figure 1A). Diabetes diagnosis was defined as fulfillment of at least 1 of 6 criteria: 1) a record of diabetes mellitus in the Clalit Chronic Disease Registry; 2) a physician’s diagnosis of diabetes with a plasma glucose test result greater than or equal to 7 mmol/L (≥126 mg/dL) within a 12-month period; 3) an HbA1c level greater than or equal to 6.5%; 4) a 2-hour plasma glucose concentration (from an oral glucose tolerance test) greater than or equal to 11 mmol/L (≥200 mg/dL); 5) 2 plasma glucose measurements greater than or equal to 7 mmol/L (≥126 mg/dL) within a 12-month period; or 6) 3 or more purchases of glucose-lowering medication (GLM) within a 12-month period. The date of diagnosis was defined as the earliest occurrence of one of these criteria. Although these definitions allowed inclusion of both type 1 and type 2 diabetes, the proportion of patients receiving insulin as their first treatment was 1.8%, indicating that over 98% of patients included in the analysis had type 2 diabetes.

Persons who were diagnosed with any cancer before 2002 (at entry into the database) were excluded, as were those who were diagnosed with prostate cancer from 2002 onward but before their diabetes diagnosis, as well as those diagnosed with prostate cancer during the first 2 years following diabetes diagnosis (before the start of follow-up—see below).

### Outcome and exposure ascertainment

Follow-up for prostate cancer was started 2 years after diabetes diagnosis. We refer to this starting point as the index date. The motivation was 1) to avoid risk of immortal time bias from having multiple criteria for diabetes diagnosis and 2) to avoid ascertainment bias (whereby diabetes is discovered while investigating symptoms caused by as-yet-undiagnosed prostate cancer) or surveillance bias (whereby prostate cancer is discovered during examinations of a patient with newly diagnosed diabetes).

Information on cancer diagnoses was obtained through linkage to the Israel Cancer Registry, as noted above. The prostate cancer outcome was identified by *International Classification of Diseases for Oncology, Third Edition*, anatomical code C61.9 and a morphology code ending in 3. The outcome date was the date of the first prostate cancer diagnosis in the registry. Individuals were followed up from their index date until the date of a prostate cancer diagnosis, death, their 90th birthday, or December 31, 2012, whichever occurred first.

Metformin exposure was defined as the purchase of a prescription for metformin, either in single pill form or as a combination pill with a dipeptidyl peptidase-4 inhibitor, although the combination form was used only from 2009 onward and comprised only 1% of all metformin purchases. We placed no restrictions on how many other glucose-lowering medications were being concurrently prescribed alongside metformin (although adjustment for other medications was made in the analysis—see “Statistical analysis” subsection).

Exposure was measured by dose, taken from the purchasing data. Dose units were determined according to defined daily dose (DDD), the assumed average maintenance dose per day for a medication used for its main indication in adults (16). Therapeutic doses for individual patients often differ from the DDD, since they are based on individual characteristics (such as age, weight, and severity of disease). The DDD provides an international standard that can be used across different studies, enhancing comparability of results.

Exposure was considered time-varying, with follow-up time split into 3-month intervals (quarter-years) and the average DDD in each interval representing metformin exposure. Further details on how this was then parameterized within the model are provided in the “Statistical analysis” subsection.

Exposure to other GLMs, including insulin, α-glucosidase inhibitors, rosiglitazone, sulfonylureas, dipeptidyl peptidase-4 inhibitors, glucagon-like peptide-1 receptor agonists, and meglitinides, was also defined according to purchase information and converted to average DDD in each 3-month interval.

While recognizing that some purchased medication may not have been taken, in the absence of information on missed medications, our analysis was based on the assumption that the amount purchased equaled the amount consumed.

### Ethical approval

The review boards of Sheba Medical Center (Ramat Gan, Israel) and Clalit Health Services approved the study proposal. The study investigators were exempted from obtaining informed consent from each patient because of the historical nature and source of the data (electronic records on a large population).

### Statistical analysis

#### Cox regression model.

We first conducted an analysis based on a Cox model with time-dependent covariates. We employed conventional discrete-time Cox regression using 3-month periods from the index date to the end of follow-up, as described above. Metformin use through time was expressed as the average dose over defined periods prior to the current one and was modeled as a weighted cumulative exposure. The method, based on that of Sylvestre and Abrahamowicz (17), is described in detail by Dankner et al. (9) and in Web Appendix 1 (available at https://doi.org/10.1093/aje/kwab287).

Because we anticipated that the relationship of metformin to prostate cancer risk would differ depending on both duration of use and recency, we divided metformin usage into 3 time windows. Specifically, we estimated the hazard ratio (HR) per average exposure (in units of DDD) during the first, second–fourth, and fifth–seventh years prior to the current 3-month period. Because these quantities were time-updated every quarter-year and the risk was only evaluated in those surviving to the current quarter, this avoided the possibility of immortal time bias that might arise from defining total duration of exposure on the basis of future information (see Figure 1B). Despite time-at-risk starting at 2 years post–diabetes diagnosis, we assessed metformin exposure (and exposure to other GLMs) in this way all the way back to the date of diabetes diagnosis, ensuring that the full duration of exposure was modeled.

We chose these time windows to distinguish periods where a possible causal association between metformin use and prostate cancer could be detected from periods of likely reverse causation or surveillance bias. Separately assessing the association with the previous year’s metformin use is important, since this period is especially susceptible to reverse causation, where, before diagnosis, prostate cancer causes a change in the prescribed metformin dosage. We also separated the period second-to-fourth years before the current quarter from the period fifth-to-seventh years before in order to distinguish between associations due to relatively recent exposure from those due to more remote exposure.

Possible confounders of the association that we adjusted for in the model included baseline age (in 5-year groups), socioeconomic status, ethnicity (see above), and exposure to other nonmetformin GLMs. The nonmetformin GLMs were grouped into 4 categories according to mechanism of action: 1) insulins (fast-acting, long-acting, intermediate-acting, and a combination of fast- and intermediate-acting); 2) medications modifying endogenic insulin levels, that is, insulin secretagogues (sulfonylureas, meglitinides) and incretin mimetics (dipeptidyl peptidase-4 inhibitors, glucagon-like peptide-1 receptor agonists); 3) α-glucosidase inhibitors; and 4) rosiglitazone, the thiazolidinedione used in Israel during the study period. The dose history of each category of GLM was represented by 3 dose variables defined in the same way as metformin. Blood glucose and HbA1c levels were not included because they were likely to be both confounders and mediators of the association between metformin and prostate cancer. To deal with this appropriately, we used a second approach—namely, IPW of an MSM (10, 18, 19).

#### IPW of an MSM.

Motivation. Previous work has shown that glucose and HbA1c levels are inversely associated with prostate cancer risk (14) and so, if not adjusted for, could result in a wrongly identified negative association between higher doses of metformin and prostate cancer. However, higher doses of metformin will also affect glucose and HbA1c levels; thus, adjustment for time-varying glucose or HbA1c level is an example of statistically adjusting for a mediator, which also introduces bias, as described in detail by Mansournia et al. (20). Such time-related confounding cannot be controlled for through standard regression modeling (21); causal methods are required. IPW of an MSM creates a weighted population in which treatment (in this case, dose of metformin) through time is independent of the time-varying confounders.

Inverse probability weights. A brief description of the analysis is presented here. Additional details are provided in Web Appendix 2.

First, a “weighting model” was constructed for estimating an individual’s probability of receiving metformin in each quarter-year of follow-up. This included all quarters from diabetes diagnosis onward to ensure that the weights appropriately reflected the probability of treatment all the way from metformin initiation to the end of follow-up. To avoid complexities with applying the analysis to continuous doses, we categorized metformin dose into 3 DDD classes: write as regular categories: 0, >0 but <0.5, or ≥0.5. Hence, we used polytomous logistic regression. Median doses in the 0, >0 but <0.5, and ≥0.5 categories were 0.25 DDD and 0.75 DDD, respectively.

The weighting model included, as covariates, quarter of follow-up, previous metformin history, and variables that were confounders of the prostate cancer–metformin relationship. Confounders included baseline HbA1c level, baseline blood glucose level, and average HbA1c and blood glucose levels over the previous 3 quarters. Since no other GLMs had been found to be associated with prostate cancer in the Cox regression analysis, we omitted them from the weighting model, to avoid positivity violations arising from including variables associated with treatment but not the outcome (22).

Second, the probability of receiving the dose received in each quarter was estimated from this model. The inverse of this, multiplied across quarters, was used to calculate the IPWs (18). We stabilized the weights by estimating a second set of weights from a model based on treatment history and time-invariant confounders alone and dividing the first set of weights by the second. To reduce positivity violations and increased variance from extreme weights (23), we truncated weights less than 0.1 and greater than 10.0. Fewer than 1% of the weights required truncation.

HbA1c values were missing for 25%–50% of patients and blood glucose values for 20%–25% of patients in any given quarter. In the context of IPW of MSMs, no clear recommendations for dealing with missing data have yet emerged. In the absence of theoretical justification for a particular method, we investigated 3 approaches: missing-value indicators, last value carried forward, and multiple imputation. Each approach has advantages and disadvantages. The missing-value indicators method leads to bias when terms for interaction between the indicator and other variables are present (24). The last-value-carried-forward method is the simplest but can lead to serious biases. Multiple imputation is valid in standard analyses and for time-invariant propensity scores, but it relies on the data being missing at random. (See Web Appendix 2 for details on each method.)

Marginal structural model. The MSM relating prostate cancer risk to medications and confounders was then fitted in the weighted population. This was done via pooled logistic regression (18) using the same quarter-year intervals as those in the discrete-time Cox model described above. For the MSM, metformin dose was modeled with the same dose categories as those used in the weighting model (0, low (>0 but <0.5), and high (≥0.5)) rather than average continuous dose. Each dose term consisted of 2 variables representing the proportion of quarters over the period in question (previous year, second–fourth years before the current quarter, or fifth–seventh years before) in which the person took low-dose or high-dose metformin, respectively. The coefficients of these 6 variables represented the log odds ratios (ORs) (which are approximately equal to the log HRs) associated with low- and high-dose metformin, respectively, in each of the 3 time periods. As in the Cox model, metformin dose was treated as time-varying, and time-invariant baseline confounders were included. In place of the Cox baseline hazard function, we included quarter as an extra factor in the logistic regression.

We also conducted an unweighted analysis of the MSM, which, like the Cox analysis, gave associations that were unadjusted for confounding caused by time-varying glucose levels. We performed this analysis to investigate the effect of the weights on the estimated associations.

#### Relating the results of the 2 models.

The Cox regression analysis yielded estimated HRs per 1 DDD of metformin per day during 3 periods (the previous year, second–fourth years before the current quarter, and fifth–seventh years before), while the MSM analysis yielded ORs for low (<0.5 DDD/day) and high (≥0.5 DDD/day) metformin dose over these periods. To compare MSM results with Cox results, we converted these ORs to a dose of 1 DDD, assuming linearity. (For details, see Web Appendix 1.)

## RESULTS

### Study population

The characteristics of the 193,835 men newly diagnosed with diabetes in 2002–2012 are shown in Table 1, alongside the subgroup of 145,617 included in our analyses (i.e., excluding those who, before or within 2 years of diabetes diagnosis, had prostate cancer diagnosed (*n* = 3,533), died (*n* = 16,049), passed the age of 90 years (*n* = 3,570), or completed follow-up (*n* = 25,066)). The number of men in this analysis subgroup diagnosed with prostate cancer on follow-up was 1,592. Their average age was 60.9 years; the largest ethnic group was Ashkenazi Jew (30.1%). Most had low or medium socioeconomic status, and approximately half were current or past smokers. Patients were followed for a median of 4.25 years (range, 0.25–9.25 years), totaling 666,553 person-years.

**Table 1:** Baseline Characteristics of All Men Insured by an Israeli Health Maintenance Organization With Incident Diabetes During 2002–2012 and Those Included in the Final Analysis

**Characteristic**	**All Men With** **Incident Diabetes** **(*n* = 193,835)**	**Men With Incident Diabetes** **Included in the Study**^**a**^ **(*n* = 145,617)**
Age at diagnosis, years^b^	60.9 (14.1)	60.9 (13.1)
Ethnic origin, %		
Ashkenazi Jew	31.7	30.1
Sephardic Jew	27.9	28.2
Israeli-born Jew	18.2	18.9
Israeli Arab	16.7	17.3
Yemenite, Ethiopian, or Central African	5.4	5.5
Socioeconomic status^c^, %		
Low	42.3	42.6
Medium	37.9	38.0
High	17.0	16.6
Missing data	2.8	2.8
Cigarette smoking, %		
Never smoker or missing data	49.5	47.3
Past smoker or current smoker	50.5	52.7

^a^ Men with incident diabetes who had at least 2 years of follow-up before prostate cancer incidence, death, or reaching age 90 years.

^b^ Values are expressed as mean (standard deviation).

^c^ Determined by locality of the Clalit Health Services clinic (Tel Aviv, Israel).

### Metformin exposure

Table 2 summarizes the GLM history of participants. Of the 95,059 men (65.3%) receiving some GLM, 75% started on metformin, receiving their first dose at a median 9 months (3 quarters) after diabetes diagnosis. They continued on metformin alone for a median 21 months (7 quarters) before switching to or adding another GLM. A subgroup of 50,558 men (34.7%), with a median 6 years of follow-up, received no GLM.

**Table 2:** Course of Glucose-Lowering Medication Use Among Israeli Men With Incident Diabetes and At Least 2 Years of Follow-up (*n* = 145,617), 2002–2012^a^

	**First Therapy**				**Second Therapy**			
			**Time From Diagnosis to Start of First Therapy, no. of quarters**				**Time From First Therapy to** **Second Therapy, no. of quarters**	
**Type of GLM**	**No. of Men**	**% of** **Subtotal**	**Mean**	**Median (IQR)**	**No. ofMen**	**% of** **Subtotal**	**Mean**	**Median (IQR)**
Metformin	71,493	75.21	6.7	3 (0–11)	7,147	19.38	9.8	7 (3–15)
Sulfonylurea	8,052	8.47	4.2	1 (0–6)	16,242	44.03	10.5	9 (4–15)
Repaglinide	2,314	2.43	7.1	3 (0–11)	6,436	17.45	11.5	10 (4–17)
Insulin	1,337	1.41	6.3	2 (0–10)	3,590	9.73	13.7	12 (6–20)
Acarbose	629	0.66	4.9	2 (0–7)	960	2.60	9.7	8 (3–14)
GLP1	27	0.03	10.0	10 (0–19)	402	1.10	15.8	14 (8–23)
DPP4i	134	0.14	15.3	13 (5–23)	1,455	3.94	14.6	14 (6–21)
Rosiglitazone	70	0.07	5.4	2.5 (0–8)	655	1.78	10.9	10 (5–16)
Metformin + sulfonylurea	6,898	7.26	4.0	0 (0–5)				
Insulin + sulfonylurea	114	0.12	3.8	0 (0–6)				
Insulin + metformin	679	0.71	5.2	1 (0–8)				
Other combination	2,377	1.63	6.0	2 (0–9)				
≥3 GLMs	935	0.64	6.3	1 (0–10)				
Subtotal	95,059		6.3	3 (0–10)	36,887		11.1	9 (4–16)
None	50,558		25.1^b^	24 (16–34)^b^	53,205		16.7^b^	15 (8–24)^b^
Total	145,617				95,059^c^			

Abbreviations: DPP4i, dipeptidyl peptidase-4 inhibitor; GLM, glucose-lowering medication; GLP1, glucagon-like peptide-1 receptor agonist; IQR, interquartile range.

^a^ Medication data were obtained for the period 1998–2011 (60 quarter-years). The study started on January 1, 2002, and follow-up continued until prostate cancer diagnosis, death, age 90 years, or December 31, 2012, whichever occurred first.

^b^ Time for “none” represents time to the end of follow-up (in quarter-years).

^c^ In addition, 4,967 men had intensification of treatment with a combination of medications or the addition of ≥1 other medications from the same group (e.g., 2 types of insulin).

### Cox regression

The estimated associations between an additional 1 DDD of metformin per day and prostate cancer risk from the time-varying Cox regression model are presented in Table 3. (Results for the full model are presented in Web Table 1.) Increased use of metformin taken over the previous year was positively associated with risk, with an estimated HR of 1.53 (95% CI: 1.19, 1.96) per additional 1 DDD. However, the estimated associations for the second–fourth years before and the second–seventh years before were negative (HR = 0.62 (95% CI: 0.41, 0.94) and HR = 0.58 (95% CI: 0.37, 0.93), respectively).

**Table 3:** Hazard Ratios for the Association of Prostate Cancer With Metformin Exposure (per 1 Defined Daily Dose per Day) in Various Time Periods During the 7 Years Prior to Cancer Diagnosis Among Israeli Men (Derived From a Cox Model), 2002–2012

**Period of Metformin Exposure** ^**a**^	HR^b^	**95% CI**
Previous year	1.53	1.19, 1.96
Second–fourth years before	0.62	0.41, 0.94
Fifth–seventh years before	0.94	0.55, 1.60
Second–seventh years before^c^	0.58	0.37, 0.93

Abbreviations: CI, confidence interval; HR, hazard ratio.

^a^ Per 1 defined daily dose of metformin per day over the specified period.

^b^ Adjusted for age (in 5-year subgroups), race/ethnicity, socioeconomic status, and history of use of other glucose-lowering medications (in 4 groups: insulins; insulin secretagogues (sulfonylureas, meglitinides) and incretin mimetics (dipeptidyl peptidase-4 inhibitors, glucagon-like peptide-1 receptor agonists); α-glucosidase inhibitors; and rosiglitazone (i.e., thiazolidinediones)).

^c^ Derived from the HRs for the second–fourth years before and the fifth–seventh years before, as follows: HR_2–7_ = HR_2–4_ × HR_5–7_.

### Marginal structural model

**Figure 2:**
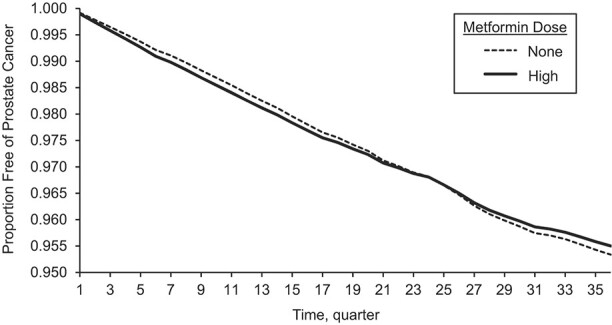
Projected prostate-cancer–free proportion of diabetic men in follow-up for 2 treatment regimens: no metformin treatment (“none”) and high-dose (≥0.5 defined daily dose) metformin treatment (“high”), Israel, 2002–2012. Estimates were based on the weighted marginal structural model using the missing-value indicators method and were computed for the age category 70–80 years, the socioeconomic status category “high,” and the ethnicity category “Ashkenazi Jew.” Follow-up extended from 2 years after diabetes diagnosis onward. Time = quarter-years of follow-up starting 2 years after diabetes diagnosis.

The weighting model output and the IPWs derived from them are described in Web Appendix 2, Web Tables 2–4, and Web Figures 1 and 2. Web Table also provides the number of person-years of follow-up and the number of events according to categories of metformin history. Table 4 presents a summary of the MSM results, showing first the results from the unweighted analysis and then the results from the weighted analyses, both using the different approaches for missing data.

**Table 4:** Odds Ratios for the Association of Prostate Cancer With Metformin Exposure (per 1 Defined Daily Dose per Day) in Various Time Periods During the 7 Years Prior to Cancer Diagnosis Among Israeli Men (Derived From Marginal Structural Models), 2002–2012

	**Unweighted MSM** ^**b**^		**Weighted MSM** ^**c**^	
**Period of Metformin Exposure** ^**a**^	**OR**	**95% CI**	**OR**	**95% CI**
*Missing-Value Indicators Method (*n *= 145,617)*				
Previous year	1.57	1.20, 2.06	1.42	1.04, 1.94
Second–fourth years before	0.65	0.41, 1.01	0.73	0.44, 1.20
Fifth–seventh years before	0.70	0.38, 1.28	0.83	0.44, 1.56
Second–seventh years before	0.45	0.26, 0.77	0.60	0.33, 1.09
*Last-Value-Carried-Forward Method (*n *= 105,412)*				
Previous year	1.41	1.05, 1.89	1.41	1.05, 1.91
Second–fourth years before	0.72	0.45, 1.16	0.77	0.48, 1.23
Fifth–seventh years before	0.62	0.33, 1.19	0.63	0.33, 1.19
Second–seventh years before	0.45	0.25, 0.80	0.48	0.27, 0.87
*Time-Sequential Imputation Method (*n *= 145,614)*				
Previous year	1.57	1.20, 2.06	1.62	1.19, 2.20
Second–fourth years before	0.65	0.41, 1.01	0.67	0.42, 1.08
Fifth–seventh years before	0.70	0.38, 1.28	0.72	0.39, 1.34
Second–seventh years before	0.45	0.27, 0.77	0.49	0.27, 0.86

Abbreviations: CI, confidence interval; OR, odds ratio; MSM, marginal structural model.

^a^ Per 1 defined daily dose of metformin per day over the specified period.

^b^ Adjusted for baseline age (in 5-year subgroups), race/ethnicity, socioeconomic status, and time since diabetes diagnosis.

^c^ Also adjusted for blood glucose level and hemoglobin A1c level (time-varying confounders).

Performing unweighted analysis, we expected results similar to those of the Cox model. Comparing these results (Table 4, columns 2 and 3) with Table 3 confirms that expectation in most respects. The OR for metformin use in the previous year (OR = 1.57, 95% CI: 1.20, 2.06) was similar to the HR from the Cox model; for the second–fourth years before, the OR and HR were also similar (OR = 0.65 vs. HR = 0.62). For the fifth–seventh years before, there was a larger difference (OR = 0.70 vs. HR = 0.94). All methods for dealing with missing data in the unweighted analysis gave similar estimates for the association between metformin and prostate cancer incidence.

Unlike the unweighted analysis, the weighted analysis adjusted for confounding by time-varying glucose control measurements. In comparison with the unweighted analysis, the ORs for metformin exposure (Table 4, columns 4 and 5) tended to move towards the null value of 1, although the direction of the point estimates for different time periods remained consistent and the 95% confidence intervals (CIs) broadly overlapped with those from the unweighted analysis. In all of the weighted analyses, the estimated positive association in the year prior to cancer diagnosis remained statistically significant (the 95% CI did not span 1); for example, for the missing indicator method, the estimated OR was 1.42 (95% CI: 1.04, 1.94). The estimated negative association in the second–seventh years before remained statistically significant in 2 analyses, but not with the missing indicator method (OR = 0.60, 95% CI: 0.33, 1.09). The 95% CIs around other estimates spanned 1. As with the unweighted analysis, the different approaches to dealing with missing data resulted in broadly consistent estimates, though it was noticeable that using the missing indicator method tended to produce the most attenuation towards the null.

Overall, adjusting for confounding by glucose control did not greatly change the results from an unweighted analysis or from the initial Cox regression model.

Finally, as a summary of the overall results from the weighted MSM analysis, we show in Figure 2 the projected prostate-cancer–free curves for 2 treatment regimens: no metformin treatment and high-dose metformin treatment (median, 0.75 DDD) from the index date onward. The figure shows a small advantage for no metformin treatment up to quarter 22 (approximately 7 years after diagnosis), with a small advantage for high-dose metformin treatment beyond that time. None of these differences were statistically significant.

## DISCUSSION

Our analysis, which accounted for major time-related biases, variations in diabetes treatment over time, and treatment modification in response to HbA1c or blood glucose level, did not support a clear relationship between metformin treatment and the risk of prostate cancer in diabetic men. Cox model results showed a positive association with recent (previous year) metformin treatment but a negative association with more distant metformin treatment (second–seventh years before). Use of IPW together with an MSM to adjust for confounding induced by glucose-level monitoring reduced the strength of these associations but did not negate them. Longer follow-up will clarify 1) whether the observed associations are confirmed and 2) whether, if confirmed, the negative association extends further back in time.

There are several advantages of using Cox regression with weighted cumulative exposure to medications to model the metformin–prostate cancer relationship. First, Cox regression with time-dependent covariates avoids the time-related biases described by Suissa et al. (8). Second, weighted cumulative exposure accounts for the complexities of time and dose in an individual’s medication history. Third, it allows adjustment for the effects of concomitant medications. A weakness in the model is the bias introduced by glucose-level monitoring as part of clinical management. If HbA1c or blood glucose levels are themselves associated with prostate cancer and are used to decide which type and level of medication to prescribe, and if in turn the medication affects these levels, a cycle of relationships is introduced that cannot be untangled by regular Cox modeling. “Causal modeling” is then required to estimate associations without bias. We have used IPW of an MSM as one such approach. This method has been used previously to assess the association between metformin monotherapy and cancer risk in diabetes patients while controlling for time-varying glucose and HbA1c levels (19), but ours is the first analysis (to our knowledge) to have combined the approach with a weighted cumulative exposure model.

Grouping the timing of therapy into the previous 1, 2–4, and 5–7 years allowed exploration of the changing association between metformin exposure and prostate cancer risk. Our finding of a positive association with metformin taken in the previous year (OR = 1.42, 95% CI: 1.04, 1.94) but a negative association with metformin taken further in the past (second–seventh years before: OR = 0.60, 95% CI: 0.33, 1.09) is open to different interpretations. The positive association may be explained by reverse causation whereby prostate cancer is already disrupting glucose control shortly before diagnosis, causing the patient to initiate metformin use or increase his dose, or by surveillance bias, whereby, before initiating metformin treatment or increasing the dose, clinicians performed more extensive checks of their patients, including prostate-specific antigen examination. If participants receiving metformin in the previous year had their prostate cancer diagnosis brought forward for one of these reasons, this could have led to fewer cases’ being associated with the metformin given in the more distant past. Alternatively, our findings may reflect a true causal association; long-term metformin use may prevent prostate cancer. Longer-term follow-up could help to settle this question, since the first explanation would lead to the waning of the association with treatment given in the more distant past, whereas a causal effect would more likely manifest itself in an association that went back as far as the latency period of the cancer.

Combined together, the positive and negative associations balance each other, leading to an overall OR of 0.86 (95% CI: 0.50, 1.47) (see Web Table 6). This estimate of overall association agrees with results from Farmer et al. (19), as well as with several recent meta-analyses. For prostate cancer, Farmer et al. estimated the overall HR for metformin therapy as 1.09 (95% CI: 0.72, 1.65) (19). The pooled HR for prostate cancer for metformin reported by Wang et al. (2) was 0.94 (95% CI: 0.79, 1.12) in 18 cohort studies. Chen et al. (1) found no association between metformin treatment and prostate cancer risk in 21 observational studies (relative risk = 1.03, 95% CI: 0.94, 1.14). Similarly, negative results were found in meta-analyses reported by He et al. (3), Feng et al. (25), and Ghiasi et al. (26).

Limitations of our study include reliance on medication purchase data for medication use; a short study duration, limiting longer-term assessment of the metformin-cancer association; limited data on some risk factors for prostate cancer, such as the use of clinic locality to determine socioeconomic status; and lack of information on other known risk factors for prostate cancer—namely family history of prostate cancer, family history of breast/ovarian cancer linked to the breast cancer 1 gene (*BRCA1*) and breast cancer 2 gene (*BRCA2*) mutations, and obesity. The strengths of our study were the large population-based cohort representative of Israeli men with diabetes in (27), assuring high external validity; high-quality data on GLM purchases and prostate cancer; and advanced analytical methods that avoided time-related biases.

Longer-term follow-up is required to clarify whether the observed negative association is confirmed and extends further back in time. Investigators with large databases of diabetic patients are encouraged to use analytical methods similar to those described here to better understand time-related associations between GLMs and chronic diseases.

## Supplementary Material

Web_Material_kwab287
