# Supplementary material for: Metformin Treatment Among Men With Diabetes and the Risk of Prostate Cancer: A Population-Based Historical Cohort Study
Source: Am J Epidemiol. 2021 Dec 10;191(4):626–35. doi: 10.1093/aje/kwab287 (PMC8971081; doi:10.1093/aje/kwab287)

# WEB MATERIAL

## Metformin Treatment Among Men With Diabetes and the Risk of Prostate Cancer: A Population-Based Historical Cohort Study

Laurence S. Freedman, Nirit Agay, Ruth Farmer, Havi Murad, Liraz Olmer, and Rachel Dankner

Table of Contents:

Web Appendices 1 and 2

Web Tables 1–6

Web Figures 1 and 2

Acknowledgements: *Unit for Biostatistics and Biomathematics, Gertner Institute for Epidemiology and Health Policy Research, Sheba Medical Center, Ramat Gan, Israel (Laurence S. Freedman, Havi Murad, Liraz Olmer); Department for Epidemiology and Preventive Medicine, School of Public Health, Sackler Faculty of Medicine, Tel Aviv University, Israel (Rachel Dankner, Laurence S. Freedman); Unit for Cardiovascular Epidemiology, The Gertner Institute for Epidemiology and Health Policy Research, Sheba Medical Center, Ramat Gan, Israel (Nirit Agay, Rachel Dankner); Department for Non Communicable Disease Epidemiology, London School of Hygiene and Tropical Medicine, London, UK* (Ruth Farmer* at the time the study was conducted).*

## Web Appendix 1

Cox Regression Model

To evaluate the association of metformin with the risk of prostate cancer among incident diabetes patients, we used a Cox regression model with time-dependent covariates. The time axis was divided into quarterly (3-month) periods, and in each period the mean daily DDD metformin level was calculated for each individual.

The model associates the risk of prostate cancer at time *t* with the weighted cumulative exposure to Metformin in the seven years before t. Specifically, these 7 years were divided into 3 periods – the year previous to *t*, years 2-4 previous to *t*, and years 5-7 previous to *t* – and in each such period we assume that the effect of metformin exposure is constant. Thus, as described in Dankner et al. (1), the weighted cumulative exposure at time *t*, denoted *WCE(t)*, is

$$WCE(t) = w_1 \sum_{u=1}^{4} D(t-u) + w_2 \sum_{u=5}^{16} D(t-u) + w_3 \sum_{u=17}^{28} D(t-u)$$

where *t* represents the current quarter, *D(t)* is the average dose taken in quarter *t*, and the *w*'s are unknown weights.

We incorporated the WCE into the Cox risk model as follows:

$$\lambda(t) = \lambda_0(t)\exp\{\beta_W WCE(t) + \beta_C C\}$$

where $\lambda(t)$ is the individual's hazard rate at time t for the cancer of interest, $\lambda_0(t)$ is the baseline hazard rate, $\beta_W$ is the coefficient of *WCE(t)*, *C* are the baseline confounders, and $\beta_C$ are their coefficients.

We get:

$$\lambda(t) = \lambda_0(t)\exp\left\{\beta_W\left[w_1\sum_{u=1}^{4}D(t-u) + w_2\sum_{u=5}^{16}D(t-u) + w_3\sum_{u=17}^{28}D(t-u) + w_4\sum_{u=29}^{40}D(t-u)\right] + \beta_C C\right\}$$

We now write:

$$D_1(t) = \sum_{u=1}^{4} D(t-u)/4\,,$$

the mean dose taken in the year previous to quarter t. Similarly, we write

$D_2(t) = \sum_{u=5}^{16} D(t-u)/12$ , and $D_3(t) = \sum_{u=17}^{28} D(t-u)/12$ , the mean doses over the years 1-4, and 5-7 previous to t.

This yields the model:

$$\lambda(t) = \lambda_0(t) exp\{\beta_W[4w_1D_1(t) + 12w_2D_2(t) + 12w_3D_3(t)] + \beta_C C\}$$

Finally, we set $\beta_1 = 4\beta_W w_1, \beta_2 = 12\beta_W w_2, \beta_3 = 12\beta_W w_{3,}$ and $\beta_4 = 12\beta_W w_4$ to give us the final Cox model used in our analysis:

$$\lambda(t) = \lambda_0(t) \exp\{\beta_1 D_1(t) + \beta_2 D_2(t) + \beta_3 D_3(t) + \beta_C C\}.$$

*$D_1(t)$*, *$D_2(t)$*, and *$D_3(t)$* are the mean daily doses over the year previous to quarter *t*, years 2-4 previous to *t*, and years 5-7 previous to *t* respectively (see Figure 1B in the main document) and can be readily calculated from the mean doses in each quarter, according to the formulas given above.

Relating the Results of the Cox and MSM Models

The Cox regression analysis yields estimates of hazard ratios per average intake 1 DDD per day of metformin during three periods (previous year, $2^{nd}$–$4^{th}$ years before and $5^{th}$–$7^{th}$ years before). However, the MSM analysis yields odds ratios associated with low (<0.5 DDD per day) and high (≥0.5 DDD per day) average metformin dose over these same periods. To compare the MSM results with the Cox regression results, we used the median doses for the low and high dose categories (0.25 DDD and 0.75 DDD respectively), and converted the odds ratios to a dose of 1 DDD assuming linearity. Specifically, if $\gamma_L$ and $\gamma_H$ denote the log odds ratios for low and high dose in a particular period, we calculated exp ($4\gamma_L$) and exp ($4\gamma_H/3$) as estimates of the odds ratios for 1 DDD increment. We considered combining these estimates in a weighted linear combination, with weights proportional to the relative precision of the two estimates. However, the precision of the estimate derived from the high dose category was so much greater than that from the low dose category that the high dose estimate completely dominated.

Therefore, we have presented the estimated odds ratio for 1 DDD derived from the high dose category. Note that due to the rarity of the prostate cancer outcome, the hazard ratio and odds ratio are almost equivalent measures of risk.

**Web Appendix 2**

Developing the Weighting Model Using Missing Value Indicators of
Glucose and HbA1c Levels

Variables Related to the Outcome (Prostate Cancer)

Since the covariates in the weighting model (WM) need to be confounders, i.e. also related to the outcome (prostate cancer), our first step was to find which covariates were indeed related to prostate cancer. These were found to be: age group, SES group, ethnic group, and year of entry to the study.

HbA1c and glucose levels were also found to be associated with prostate cancer (2). Our main enquiry was to find out which HbA1c or glucose measurements were related to current prostate cancer risk. For this purpose, we ran a series of Cox regressions, each one including a longer history of HbA1C/glucose levels. Each HbA1c or glucose term included two variables –a continuous variable representing the level in that quarter, and a binary variable indicating a missing (or not) level for that quarter. By comparing the log likelihood of these models we decided to include the HbA1C and glucose variables of three previous quarters.

Now we were ready to construct a model predicting the treatment category in each quarter t (No Metformin, Low dose, or High dose). First, we ran a series of polytomous logistic regression models for the following quarters: 10, 15, 20, 40 (following diabetes diagnosis). After comparing many different models, we decided to use the mean of the HbA1C levels in the three previous quarters, and one missing indicator denoting if all three were missing. The same variables for glucose levels were also included. Therefore, the variables in the WM were chosen to be the following: age group, SES group, ethnicity group, year of entry, the mean HbA1C level in the three previous quarters with a missing indicator and the mean glucose level in the three previous quarters with a missing indicator, and importantly, the doses of metformin (non, low, high) in the four previous quarters.

There was a considerable amount of missing data: the mean HbA1c averaged over the past 3 quarters could not be calculated for 25-50% of patients (depending on the quarter) and the mean glucose could not be calculated for about 20-25% of patients. In addition, missing HbA1c was found to be clearly related to glucose level. It seems that clinicians tended to order a HbA1c test according to the glucose level. If the glucose level was higher, they were more likely to measure HbA1c.

So, our next step was to examine the interactions between the missing HbA1c/glucose indicators and other variables, and to determine which interactions should enter the model. We decided to retain in the model only those that were (a) statistically significant at the two-sided 5% level, and (b) had absolute value greater than 0.4 for binary variable interactions or whose absolute value times the interquartile range of the variable was greater than 0.4. This condition corresponded to an interaction which changed small probabilities by 50% or more, e.g. 0.03

changed to 0.045 (exp(ln(0.03)+0.4)) or more. Finally, we included metformin treatment history: the treatment category in each one of the previous four quarters. We ended up with 2 models, one for quarters 1-22 another for quarters 23-44.

We then built a “pooled model” over all quarters. This was a multinomial logistic model with each quarter for each individual treated as an independent observation. This was done using the Logistic Procedure in SAS with the “dist=multinomial” option, specifying the reference category in the outcome to be the null dose of metformin. In other words, the outcome variable was two-dimensional: receiving low-dose metformin compared to none, and receiving high-dose metformin compared to none. The pooled model included a binary “time” variable, denoting whether the quarter is below 23, and its interaction terms with some of the other variables (we explored which of those were important). This pooled weighting model and its parameter estimates are shown in the Model 1 columns of Web Table 2 (end of document).

From this pooled model we obtained, for each patient and for each quarter, the probability of receiving no dose, low dose or high dose of metformin. The weights for each individual are then calculated as the inverse of the probability of receiving the observed dose, cumulated over the quarters. These weights become very large and require “stabilization” for good performance. They were stabilized by running the same WM but without the HBA1c and glucose variables, obtaining the cumulative probabilities also from this model, and their corresponding cumulative weights, and then dividing the cumulative weights from the full WM by those from the “stabilizing” model.

The distribution of the resulting weights is shown in Web Figure 1 for three selected quarters, and percentiles are shown in Web Table 3. We truncated the weights at 0.1 and 10, and then ran the outcome model with these weights (as detailed in the main text).
To check on the stability of our results, we explored changing the truncation rule for the weights. Instead of truncating at 0.1 and 10, we explored truncating at 0.05 and 20, and at 0.2 and 5. Second, we tried different parameterizations of time – a common intercept for all quarters, and a linear trend over quarters. The estimated parameters describing the association of metformin with prostate cancer were stable across these sensitivity analyses.

<u>Last Value Carried Forward (LVCF)</u>

An alternative method for dealing with the missing values of HbA1C and glucose levels was to impute them via Last Value Carried Forward rule. In order to implement this method, we needed to include only those patients with known baseline values of HbA1c and blood glucose. We defined this as having at least one measurement of HbA1c and one measurement of blood glucose in the first 8 quarters following diabetes diagnosis. This condition was fulfilled in 72% of the population. For this subgroup, data can be imputed via LVCF and the weighting model becomes simpler (there are no missing value indicators and no interactions, and also the binary "time" variable is unnecessary).

The weighting model and estimated parameters is shown in the Model 2 columns of Web

Table 2 and the distribution of weights for selected quarters in Web Figure 2 and Web Table 4 (see end of document).

To compare the results of LVCF analysis with those of the Missing Value Indicator analysis, we restricted the Missing Value Indicator analysis to the same 72% of the sample. All the associations were somewhat weaker than those estimated under LVCF. The negative association with taking metformin over the 2-7 years previously was weaker than estimated under LVCF, and the 95% CI for the OR included 1.0. (Results not shown here.)

Multiple Imputation

The third method for dealing with the missing HbA1C and glucose levels was multiple imputation.

Our PS model predicts the treatment in quarter t according to the mean of the three previous HBA1C measurements, and the mean of the three previous glucose measurements. We therefore imputed all the missing measurements and then for each quarter computed the mean. We used the sequential imputation method described in Murad et al. (3).

We imputed missing log(glucose) and log(HbA1c) in quarter t iteratively in a chain. The imputation model for log(glucose) in quarter t included log(HbA1c) in quarter t (imputed if missing), and also log (glucose) and log (HbA1c) in quarters t-1 and t-2 (imputed if missing), medication in quarters t+1, t+2, t+3, SES, age group, year of entry, ethnic origin, and an indicator for cancer in t+2, t+3, t+4 (diagnosed/not diagnosed). If follow-up ended in one of the three future quarters (t+1, t+2, t+3), then medications in the quarters after follow-up ended were set to 0. We started the imputation at quarter 1 and proceeded sequentially to quarter 43. The imputation model for log(HbA1c) in quarter t was similar. Five sets of imputations were used. For each imputation set, the PSM parameters were estimated and the inverse probability weights obtained. Then the pooled logistic regression model was run and the parameters of the model estimated. Final estimates of the parameters and their standard errors were obtained using Rubin’s rules. Results are shown in Table 4 of the main text and in Web Table 6, and are similar to those of the “naïve” unweighted analysis.

Person-Years and Events for Metformin History Categories

The marginal structural model (MSM) relates prostate cancer risk to metformin exposure history in the form of three categories of metformin dose (zero, low, high) used in three different periods (previous 1, 2-4 and 5-7 years). This gives rise to 27 (3x3x3) different category combinations describing metformin history (three possible levels in the previous year, three levels in years 2-4 before, and three levels in years 5-7 before). As basic information underlying the MSM analysis, we provide in Web Table 5 the number of person years of observation and the number of events occurring for each of these 27 category combinations. It can be seen from the table that the most commonly observed combinations, having more than 20,000 person years and more than 50

prostate cancer events, are: (i) no metformin medication, (ii) low dose for the past year and none before that, (iii) low dose for the past 4 years and none before that, (iv) high dose for the past year and none before that, (v) high dose for the past 4 years and none before that, and (vi) high dose for the past year, low dose in the 3 years before that and none before that.

## References


1. Dankner R, Agay N, Olmer L, et al. Metformin treatment and cancer risk: Cox regression analysis, with time-dependent covariates, of 320,000 persons with incident diabetes mellitus. *Am J Epidemiol.* 2019;188(10):1794-1800.
2. Dankner R, Boker LK, Boffetta P, et al. A historical cohort study on glycemic-control and cancer-risk among patients with diabetes. *Cancer Epidemiol.* 2018;57:104-109.
3. Murad H, Dankner R, Berlin A, et al. Imputing missing time-dependent covariate values for the discrete time Cox model. *Stat Methods Med Res.* 2020;29(8):2074–2086.

Web Table 1. More detailed results from the Cox model for association of prostate cancer with metformin exposure (DDD) in the 7 previous years

| Variable | Cox model | |
|---|---|---|
| | HR | 95% CI |
| Time of metformin exposure [a] | | |
| Previous year | 1.53 | 1.19, 1.96 |
| 2nd – 4th years before | 0.62 | 0.41, 0.94 |
| 5th – 7th years before | 0.94 | 0.55, 1.60 |
| Previous 7 years [b] | 0.89 | 0.58, 1.35 |
| 2nd – 7th years before [c] | 0.58 | 0.37, 0.93 |
| Age, years | | |
| Reference category: 21 to 50 | 1.00 | - |
| 50 – 60 | 8.83 | 5.66, 13.78 |
| 60 – 70 | 25.26 | 16.34, 39.05 |
| 70 – 80 | 28.58 | 18.44, 44.29 |
| 80 – 90 | 26.48 | 16.68, 42.03 |
| SES | | |
| Reference category: Low | 1.00 | - |
| High | 1.27 | 1.10, 1.46 |
| Medium | 1.13 | 1.00, 1.27 |
| Missing | 1.01 | 0.74, 1.39 |
| Ethnic background | | |
| Reference category: Ashkenazi Jewish | 1.00 | - |
| Ethiopian Jewish | 0.48 | 0.25, 0.92 |
| Arab | 0.57 | 0.46, 0.71 |
| Yemenite Jewish | 0.64 | 0.48, 0.85 |
| Israeli Born Jewish | 0.89 | 0.77, 1.04 |
| Sephardi Jewish | 1.05 | 0.94, 1.19 |
| Time of exposure to insulins [a] | | |
| Previous year | 0.62 | 0.31, 1.25 |
| 2nd – 4th years before | 1.44 | 0.48, 4.37 |
| 5th – 7th years before | 0.75 | 0.12, 4.86 |
| Time of exposure to endogenics [d,a] | | |
| Previous year | 0.99 | 0.72, 1.35 |
| 2nd – 4th years before | 1.08 | 0.61, 1.89 |
| 5th – 7th years before | 0.70 | 0.31, 1.60 |
| Time of exposure to α-glucosidase inhibitors [a] | | |
| Previous year | 1.37 | 0.14, 14.0 |
| 2nd – 4th years before | 0.25 | 0.00, 16.7 |
| 5th – 7th years before | 2.49 | 0.02, 286 |
| Time of exposure to rosiglitazone [a] | | |
| Previous year | 0.08 | 0.00, 1.85 |
| 2nd – 4th years before | 1.84 | 0.10, 32.5 |
| 5th – 7th years before | 0.22 | 0.00, 91.8 |

Abbreviations: DDD, Defined Daily Dose; CI, confidence interval; HR, hazard ratio; SES socioeconomic status.

[a] Per increase of 1 DDD per day. [b] Derived from previous year HR, 2nd–4th year HR and 5th–7th year HR as follows: HR(1-7) = HR(1) x HR(2-4) x HR(5-7); [c] Derived from 2nd–4th year HR and 5th–7th year HR as follows: HR(2-7) = HR(2-4) x HR(5-7). [d] (sulfonylureas, repaglinide, DPP4- inhibitor, GLP1–receptor agonist).

Web Table 2. The Pooled Weighting Models; Model 1 Using Missing Indicator Variables; Model 2 Using LVCF: A Multivariate Logistic Model for the Probability to Receive a Low/High Dose of Metformin, Compared to None

| Variable | Category | Metformin dose level vs none | Model 1 | | Model 2 | |
|---|---|---|---|---|---|---|
| | | | β | se | β | se |
| Year of entry to the study (2002-2010 coded as 1-9) | | Low | -0.012 | 0.009 | -0.004 | 0.001 |
| | | High | -0.043 | 0.009 | -0.026 | 0.001 |
| Age group (reference: >70) | 21-40 | Low | -0.158 | 0.029 | -0.081 | 0.013 |
| | | High | -0.050 | 0.033 | 0.014 | 0.016 |
| | 40-55 | Low | 0.119 | 0.020 | 0.141 | 0.008 |
| | | High | 0.300 | 0.022 | 0.294 | 0.010 |
| | 55-70 | Low | 0.176 | 0.019 | 0.208 | 0.008 |
| | | High | 0.377 | 0.021 | 0.361 | 0.009 |
| Socio-economic status (reference: Low) | High | Low | 0.012 | 0.008 | -0.007 | 0.009 |
| | | High | 0.144 | 0.009 | 0.119 | 0.010 |
| | Medium | Low | 0.032 | 0.006 | 0.016 | 0.006 |
| | | High | 0.087 | 0.007 | 0.071 | 0.008 |
| | Missing | Low | 0.022 | 0.015 | 0.019 | 0.017 |
| | | High | 0.061 | 0.018 | 0.058 | 0.020 |
| Ethnic origin (reference: Ashkenazi) | Arab | Low | 0.086 | 0.008 | 0.059 | 0.009 |
| | | High | -0.178 | 0.009 | -0.194 | 0.010 |
| | Ethiopian | Low | 0.065 | 0.021 | 0.082 | 0.023 |
| | | High | -0.200 | 0.026 | -0.171 | 0.028 |
| | Israeli | Low | 0.044 | 0.008 | 0.016 | 0.009 |
| | | High | 0.001 | 0.009 | -0.016 | 0.010 |
| | Sephardic | Low | 0.055 | 0.007 | 0.037 | 0.008 |
| | | High | -0.081 | 0.008 | -0.095 | 0.009 |
| | Yemenite | Low | 0.029 | 0.013 | 0.014 | 0.014 |
| | | High | -0.100 | 0.015 | -0.107 | 0.016 |
| Mean HbA1c in 3 previous quarters | | Low | 0.056 | 0.003 | 0.051 | 0.003 |
| | | High | 0.141 | 0.003 | 0.115 | 0.003 |
| Missing HbA1c | | Low | -1.788 | 0.028 | 0.001 | 0.000 |
| | | High | -2.105 | 0.034 | 0.002 | 0.000 |
| Mean glucose in 3 previous quarters | | Low | 0.001 | 0.000 | -0.009 | 0.002 |
| | | High | 0.001 | 0.000 | -0.006 | 0.002 |
| Missing glucose | | Low | 0.096 | 0.012 | 0.000 | 0.000 |
| | | High | 0.088 | 0.015 | -0.001 | 0.000 |
| Missing HbA1c* Mean glucose | | Low | 0.009 | 0.000 | -0.070 | 0.009 |
| | | High | 0.011 | 0.000 | -0.091 | 0.010 |
| Missing HbA1c *Year of entry | | Low | -0.102 | 0.016 | -0.149 | 0.020 |
| | | High | -0.081 | 0.020 | -0.182 | 0.023 |
| Missing HbA1c *Missing glucose*Year of entry | | Low | 0.165 | 0.018 | 2.462 | 0.007 |
| | | High | 0.189 | 0.021 | 2.598 | 0.010 |
| Time (1 if quarter<23) | | Low | 0.115 | 0.022 | 2.285 | 0.010 |
| | | High | 0.072 | 0.025 | 3.796 | 0.011 |
| Missing HbA1c *Year of entry*Time | | Low | 0.050 | 0.016 | 1.320 | 0.008 |
| | | High | 0.029 | 0.019 | 1.140 | 0.010 |
| Missing HbA1c *missing glucose*Year of entry*Time | | Low | -0.109 | 0.017 | 1.062 | 0.011 |
| | | High | -0.128 | 0.021 | 2.095 | 0.012 |

| Variable | Category | Metformin dose level vs none | Model 1 | | Model 2 | |
|---|---|---|---|---|---|---|
| | | | β | se | β | se |
| Time*Age group | 21-40 | Low<br>High | 0.015<br>-0.017 | 0.031<br>0.036 | 0.840<br>0.620 | 0.008<br>0.010 |
| Time*Age group | 40-55 | Low<br>High | 0.009<br>-0.045 | 0.021<br>0.024 | 0.496<br>1.166 | 0.012<br>0.012 |
| Time*Age group | 55-70 | Low<br>High | 0.029<br>-0.049 | 0.021<br>0.023 | 0.752<br>0.575 | 0.008<br>0.010 |
| Time*year of entry | | Low<br>High | 0.008<br>0.024 | 0.009<br>0.009 | 0.409<br>0.984 | 0.011<br>0.012 |
| Low dose of Metformin in previous quarter | | Low<br>High | 2.531<br>2.653 | 0.006<br>0.009 | | |
| High dose of Metformin in previous quarter | | Low<br>High | 2.340<br>3.825 | 0.009<br>0.010 | | |
| Low dose of Metformin 2 quarters before | | Low<br>High | 1.347<br>1.165 | 0.007<br>0.009 | | |
| High dose of Metformin 2 quarters before | | Low<br>High | 1.072<br>2.101 | 0.010<br>0.011 | | |
| Low dose of Metformin 3 quarters before | | Low<br>High | 0.863<br>0.645 | 0.008<br>0.010 | | |
| High dose of Metformin 3 quarters before | | Low<br>High | 0.497<br>1.168 | 0.011<br>0.012 | | |
| Low dose of Metformin 4 quarters before | | Low<br>High | 0.799<br>0.637 | 0.007<br>0.009 | | |
| High dose of Metformin 4 quarters before | | Low<br>High | 0.455<br>1.039 | 0.010<br>0.011 | | |

Web Table 3. Weights Distribution for Selected Quarters Derived From the Weighting Model with Missing Indicators (After Truncation)

| Percentile | Weight2 | Weight16 | Weight34 |
|---|---|---|---|
| 99% | 1.49 | 2.96 | 5.37 |
| 90% | 1.05 | 1.31 | 1.61 |
| 75% | 1.02 | 1.09 | 1.16 |
| 50% | 0.99 | 0.92 | 0.86 |
| 25% | 0.96 | 0.75 | 0.64 |
| 10% | 0.93 | 0.63 | 0.47 |
| 1% | 0.65 | 0.34 | 0.23 |

Web Table 4. Weights Distribution for Selected Quarters Derived from the Weighting Model Using Last Value Carried Forward (After Truncation)

| Percentile | Weight2 | Weight16 | Weight34 |
|---|---|---|---|
| 99% | 1.21 | 1.86 | 2.79 |
| 90% | 1.03 | 1.18 | 1.35 |
| 75% | 1.00 | 1.04 | 1.09 |
| 50% | 1.00 | 0.97 | 0.95 |
| 25% | 0.99 | 0.90 | 0.83 |
| 10% | 0.96 | 0.82 | 0.71 |
| 1% | 0.79 | 0.57 | 0.48 |

Web Table 5. Person Years of Observation and Number of Events Observed According to Metformin History

| Metformin dose in previous year | Metformin dose in previous 2-4y | Metformin dose in previous 5-7y | Person years of observation | Number of prostate cancer events |
|---|---|---|---|---|
| High | High | High | 17,630 | 35 |
| High | High | Low | 11,792 | 28 |
| High | High | Zero | 37,564 | 90 |
| High | Low | High | 2,656 | 5 |
| High | Low | Low | 6,315 | 16 |
| High | Low | Zero | 23,488 | 62 |
| High | Zero | High | 884 | 2 |
| High | Zero | Low | 1532 | 4 |
| High | Zero | Zero | 38,650 | 92 |
| Low | High | High | 2,411 | 4 |
| Low | High | Low | 2,716 | 5 |
| Low | High | Zero | 9,909 | 20 |
| Low | Low | High | 1,799 | 3 |
| Low | Low | Low | 7,676 | 17 |
| Low | Low | Zero | 34,728 | 86 |
| Low | Zero | High | 643 | 1 |
| Low | Zero | Low | 2,021 | 4 |
| Low | Zero | Zero | 57,910 | 134 |
| Zero | High | High | 1,690 | 1 |
| Zero | High | Low | 1,439 | 1 |
| Zero | High | Zero | 5,929 | 9 |
| Zero | Low | High | 930 | 1 |
| Zero | Low | Low | 2,692 | 4 |
| Zero | Low | Zero | 16,630 | 28 |
| Zero | Zero | High | 2,438 | 3 |
| Zero | Zero | Low | 5,923 | 12 |
| Zero | Zero | Zero | 368,558 | 925 |
| **Total** | | | **666,553** | **1,592** |

Web Table 6. More Details of the Results Derived from the Marginal Structural Model: Odds Ratios for the Association of Prostate Cancer with Metformin Exposure

| | Marginal Structural Model | | | |
|---|---|---|---|---|
| | Unweighted | | Weighted | |
| Variable | *OR* | *95% CI* | *HR* | *95% CI* |
| *Missing Value Indicator Method (n* = 145,617*)* | | | | |
| Metformin exposure [a] | | | | |
| Previous year | 1.57 | 1.20, 2.06 | 1.42 | 1.04, 1.94 |
| 2nd – 4th years before | 0.65 | 0.41, 1.01 | 0.73 | 0.44, 1.20 |
| 5th – 7th years before | 0.70 | 0.38, 1.28 | 0.83 | 0.44, 1.56 |
| Previous 7 years [b] | 0.71 | 0.43, 1.17 | 0.86 | 0.50, 1.47 |
| 2nd – 7th years before [c] | 0.45 | 0.26, 0.77 | 0.60 | 0.33, 1.09 |
| Age, years | | | | |
| Ref. category: 21 to 50 | 1.00 | - | 1.00 | - |
| 50 – 60 | 8.92 | 5.71, 13.93 | 10.64 | 6.75, 16.77 |
| 60 – 70 | 25.65 | 15.59, 39.67 | 32.17 | 20.63, 50.17 |
| 70 – 80 | 29.02 | 18.71, 45.02 | 36.81 | 23.54, 57.58 |
| 80 – 90 | 26.78 | 16.86, 42.54 | 43.18 | 21.18, 55.18 |
| SES | | | | |
| Ref. category: Low | 1.00 | - | 1.00 | - |
| High | 1.27 | 1.10, 1.46 | 1.23 | 1.06, 1.43 |
| Medium | 1.13 | 1.01, 1.27 | 1.12 | 0.99, 1.27 |
| Missing | 1.01 | 0.74, 1.39 | 0.99 | 0.72, 1.37 |
| Ethnicity | | | | |
| Ref. category: Ashkenazi Jewish | 1.00 | - | 1.00 | - |
| Ethiopian Jewish | 0.48 | 0.25, 0.92 | 0.50 | 0.25, 0.98 |
| Arab | 0.57 | 0.46, 0.70 | 0.55 | 0.44, 0.69 |
| Yemenite Jewish | 0.64 | 0.48, 0.85 | 0.61 | 0.45, 0.82 |
| Israeli born Jewish | 0.90 | 0.77, 1.04 | 0.92 | 0.78, 1.08 |
| Sephardi Jewish | 1.05 | 0.94, 1.19 | 1.06 | 0.93, 1.20 |
| *Last Value Carried Forward Method (n* = 105,412*)* | | | | |
| Metformin exposure [a] | | | | |
| Previous year | 1.41 | 1.05, 1.89 | 1.41 | 1.05, 1.91 |
| 2nd – 4th years before | 0.72 | 0.45, 1.16 | 0.77 | 0.48, 1.23 |
| 2nd – 7th years before [c] | 0.45 | 0.25, 0.80 | 0.48 | 0.27, 0.87 |
| *Time-sequential Imputation Method (n* = 145,614*)* | | | | |
| Metformin exposure [a] | | | | |
| Previous year | 1.57 | 1.20, 2.06 | 1.62 | 1.19, 2.20 |
| 2nd – 4th years before | 0.65 | 0.41, 1.01 | 0.67 | 0.42, 1.08 |
| 2nd – 7th years before [c] | 0.45 | 0.27, 0.77 | 0.49 | 0.27, 0.86 |

Abbreviations: DDD, defined daily dose; CI, confidence interval; HR, hazard ratio; Ref., Reference; SES, socioeconomic status

[a] Exposure to 1 DDD per day of metformin over the specified period

[b] Derived from previous year HR, 2nd–4th years before HR and 5th–7th years before HR as follows: $HR_{1\text{-}7} = HR_1 \times HR_{2\text{-}4} \times HR_{5\text{-}7}$;

[c] Derived from 2nd–4th years before HR and 5th–7th years before HR as follows: $HR_{2\text{-}7} = HR_{2\text{-}4} \times HR_{5\text{-}7}$.

Web Figure 1.  Weights Distribution for Selected Quarters Derived from the Weighting Model with Missing Indicators (After Truncation)

Weights for quarter 2, which is the 10th quarter since diabetes diagnosis

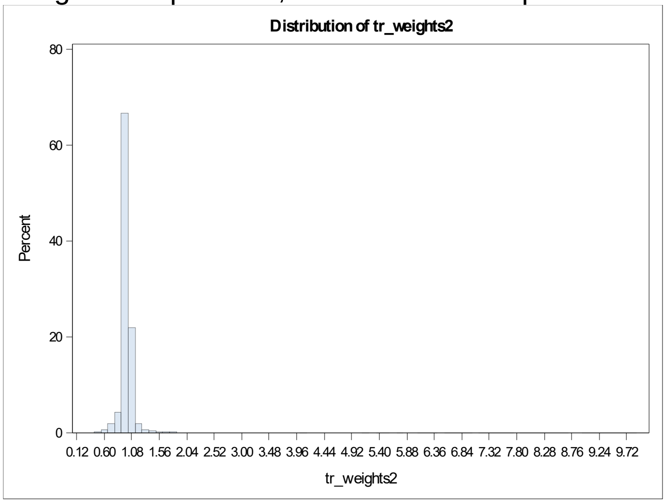


Weights for quarter 16, which is the 24th quarter since diabetes diagnosis

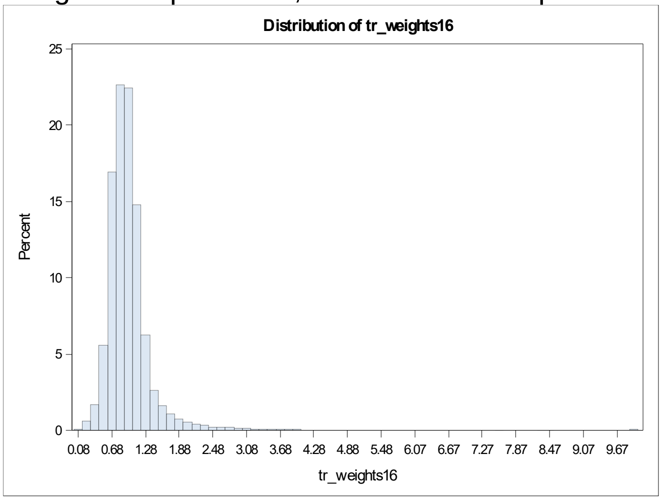


Weights for quarter 34, which is the 42nd quarter since diabetes diagnosis

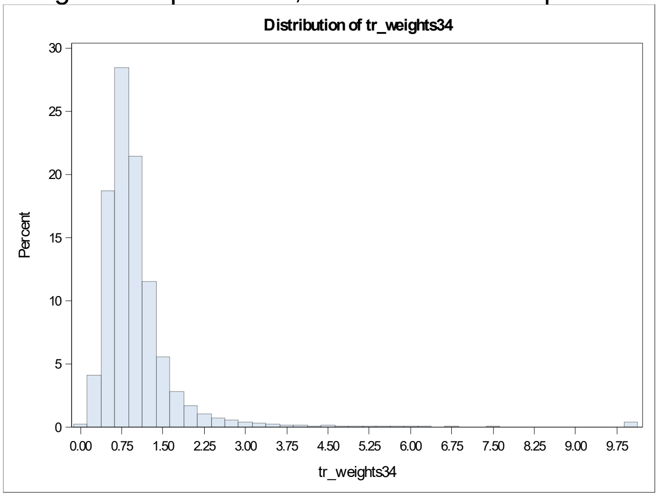

Web Figure 2. Weights Distribution for Selected Quarters Derived from the Weighting Model Using Last Value Carried Forward (After Truncation)

Weights for quarter 2, which is the 10th quarter since diabetes diagnosis

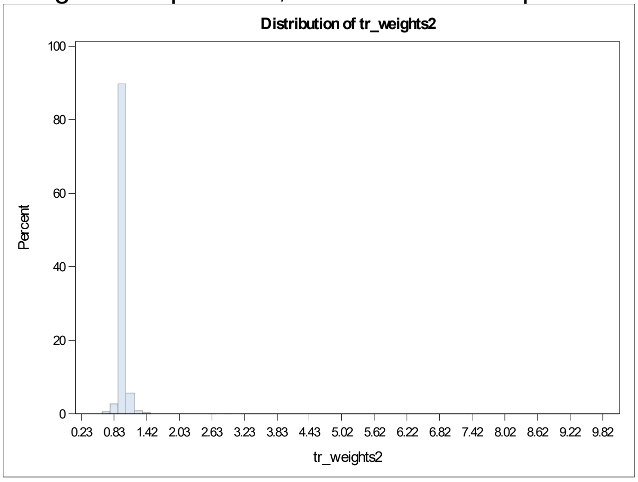


Weights for quarter 16, which is the 24th quarter since diabetes diagnosis

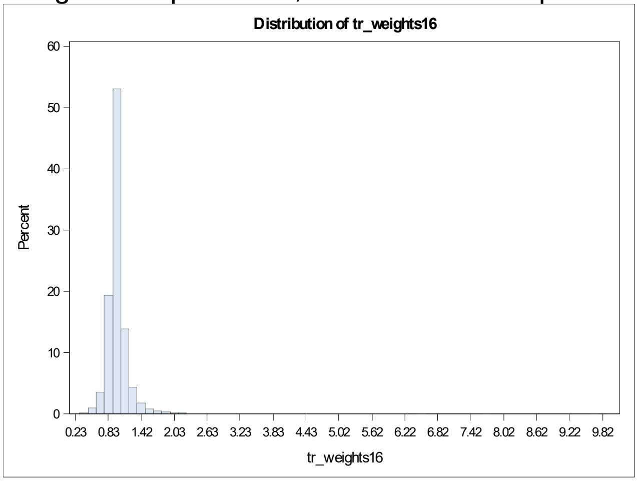


Weights for quarter 34, which is the 42nd quarter since diabetes diagnosis

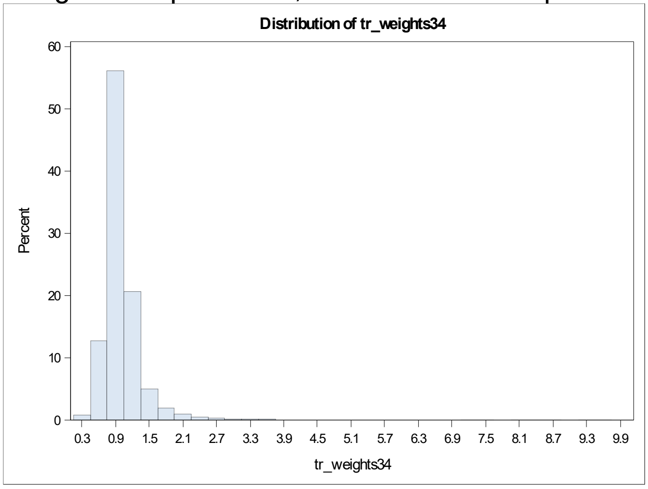

Supplement: Web_Material_kwab287 [file web_material_kwab287.pdf]
